# Conformational Rigidity within Plasticity Promotes Differential Target Recognition of Nerve Growth Factor

**DOI:** 10.3389/fmolb.2016.00083

**Published:** 2016-12-26

**Authors:** Francesca Paoletti, Cesira de Chiara, Geoff Kelly, Sonia Covaceuszach, Francesca Malerba, Robert Yan, Doriano Lamba, Antonino Cattaneo, Annalisa Pastore

**Affiliations:** ^1^Neurotrophic Factors and Neurodegenerative Diseases Unit, European Brain Research, Rita Levi-Montalcini FoundationRome, Italy; ^2^Scuola Normale SuperiorePisa, Italy; ^3^The Francis Crick InstituteLondon, UK; ^4^Medical Research Council (MRC) Biomedical NMR Centre, The Francis Crick InstituteLondon, UK; ^5^Istituto di Cristallografia, Consiglio Nazionale delle Ricerche (CNR), Sede Secondaria di BasovizzaTrieste, Italy; ^6^Maurice Wohl Institute, Department of Basic and Clinical Neuroscience, King's College LondonLondon, UK; ^7^Molecular Medicine Department, University of PaviaPavia, Italy

**Keywords:** antibody recognition, NGF, neurodegeneration, neurotrophins, NMR, structure

## Abstract

Nerve Growth Factor (NGF), the prototype of the neurotrophin family, is essential for maintenance and growth of different neuronal populations. The X-ray crystal structure of NGF has been known since the early '90s and shows a β-sandwich fold with extensive loops that are involved in the interaction with its binding partners. Understanding the dynamical properties of these loops is thus important for molecular recognition. We present here a combined solution NMR/molecular dynamics study which addresses the question of whether and how much the long loops of NGF are flexible and describes the N-terminal intrinsic conformational tendency of the unbound NGF molecule. NMR titration experiments allowed identification of a previously undetected epitope of the anti-NGF antagonist antibody αD11 which will be of crucial importance for future drug lead discovery. The present study thus recapitulates all the available structural information and unveils the conformational versatility of the relatively rigid NGF loops upon functional ligand binding.

## Introduction

Nerve Growth Factor (NGF), the prototype of the neurotrophin family, is essential for maintenance and growth of different neuronal populations in the nervous systems (Levi-Montalcini, 1987). Alterations in its homeostatic regulation are involved in severe pathologies (Tiveron et al., 2013; Mysona et al., 2015). NGF is translated as a precursor, proNGF, with distinct biological functions (Hempstead, 2014). An early event in NGF signal transduction is the interaction with p75^NTR^ and/or TrkA receptors (Lewin and Carter, 2014). Because of its role in the pathway of pain (Capsoni, 2014; Kelleher et al., 2017), there is a substantial interest in developing NGF mimetics endowed with antagonistic properties. However, this strongly relies on a detailed knowledge of its structure and of the interactions with its receptors.

The X-ray crystal structures of NGF have been known since the early 90s (McDonald et al., 1991; Holland et al., 1994). NGF is an obligate non-covalent head-to-head homodimer with three loops (I, II, and V) at one end of the molecule, whereas the opposite end contains a loop structure (III) and the conserved cystine-knot arrangement of the three intra-chain disulphide bonds. A distinctive feature of NGF are its long unstructured loops (I, II, III, and V), important for its interactions with the binding partners.

Also available are the structures of NGF bound to its receptors (Wiesmann et al., 1999; He and Garcia, 2004; Wehrman et al., 2007; Feng et al., 2010), neutralizing antibodies (Covaceuszach et al., 2008; La Porte et al., 2014), and small ligands, such as lysophospholipids (Tong et al., 2012; Sun and Jiang, 2015) and DNA-aptamers (Jarvis et al., 2015). These studies have led to an emerging picture of the binding determinants on NGF to its key receptors.

Despite the wealth of structural information obtained from the extensive crystallographic investigations, some significant questions have remained elusive: (i) is the NGF N-terminus unstructured in the absence of ligands or does it already have some intrinsic conformational tendency? (ii) how flexible/rigid are the loops and how their dynamics may reflect on the overall structural plasticity of mature NGF? (iii) how much do the loops contribute to antibody recognition?

Structural information is thus extremely important, in view of structure-based design of effective non-peptidic antagonists of NGF activity on TrkA receptor. Accumulating evidence shows that the NGF N-terminus plays a role in a number of processes. An N-terminal truncated form of NGF has a significant drop in affinity for TrkA and in the ability of eliciting TrkA phosphorylation (Kahle et al., 1992; Hughes et al., 2001) which could not be ascribed to differences in folding or stability (Woo et al., 1995). In one of the two crystal structures of NGF purified from mouse salivary glands (mNGF), the N-terminus is absent (Holland et al., 1994) whereas in the other neither N- nor C- termini were defined (McDonald et al., 1991). The N-terminus in recombinant human NGF (hNGF) or mouse proNGF in complex with p75^NTR^ are not defined (He and Garcia, 2004; Feng et al., 2010). Only in the hNGF/TrkA complex (Wiesmann et al., 1999; Wehrman et al., 2007) and in one of the two protomers of a complex with a DNA aptamer (Jarvis et al., 2015), is the N-terminus defined and folded in an α-helix. The generally accepted view is thus that the N-terminus is disordered in the NGF unbound state (Settanni et al., 2003; Berrera et al., 2006). Similar conclusions have been recently reached as result of CD and NMR solution studies and molecular dynamics simulations on two linear hNGF N-terminus peptides (Stanzione et al., 2010; Travaglia et al., 2015).

It is difficult to infer from the X-ray data whether the variations reflect authentic flexibility or rather a structural plasticity. The two terms are similar but not equivalent: In the context of a protein, we would consider plasticity the capacity of a region to change the conformation permanently under, for instance, an interaction; flexibility is the property of a region (e.g., a loop) not to have a fixed conformation. In other words, one is a static concept, the second dynamic. The difference is particularly important for the design of antibodies directed against specific regions of NGF. Among the existing anti-NGF antibodies, the well-characterized therapeutic MAb αD11 (Cattaneo et al., 1988; Covaceuszach et al., 2012) is particularly interesting as a structural probe, because it recognizes preferentially the mature form of NGF vs. proNGF (Paoletti et al., 2009). This property has been used to study the mechanistic consequences of inducing experimentally an imbalance in NGF/proNGF (Capsoni and Cattaneo, 2006; Capsoni et al., 2011). The MAb αD11 epitope has been characterized by ELISA, and the structure of the complex of rat αD11FAb and hNGF was obtained by *in silico* computational docking and validated by SAXS experiments (Covaceuszach et al., 2008).

We present here a study in solution which addresses the open questions. Using a combination of nuclear magnetic resonance (NMR) and molecular dynamics (MD), we describe the N-terminal intrinsic conformational preferences of unbound NGF in solution. We also show that in the absence of partners the NGF N-terminus has a strong tendency to fold into a helix, challenging the current view that this region is unstructured. Our study also sets a definitive word on the structural plasticity of NGF loops II and V and provides a structural explanation for the large differential affinity of the αD11 anti-NGF therapeutic antibody for NGF vs. proNGF. We demonstrate by solution NMR epitope mapping with the MAb αD11 the presence of a previously undetected epitope. The present study thus fills a gap in our structural understanding of NGF inter- and intra-molecular interactions and provides a strong basis for the design of more selective NGF antagonists.

## Results

### Solution NMR structure of mouse NGF

**Figure 1:**
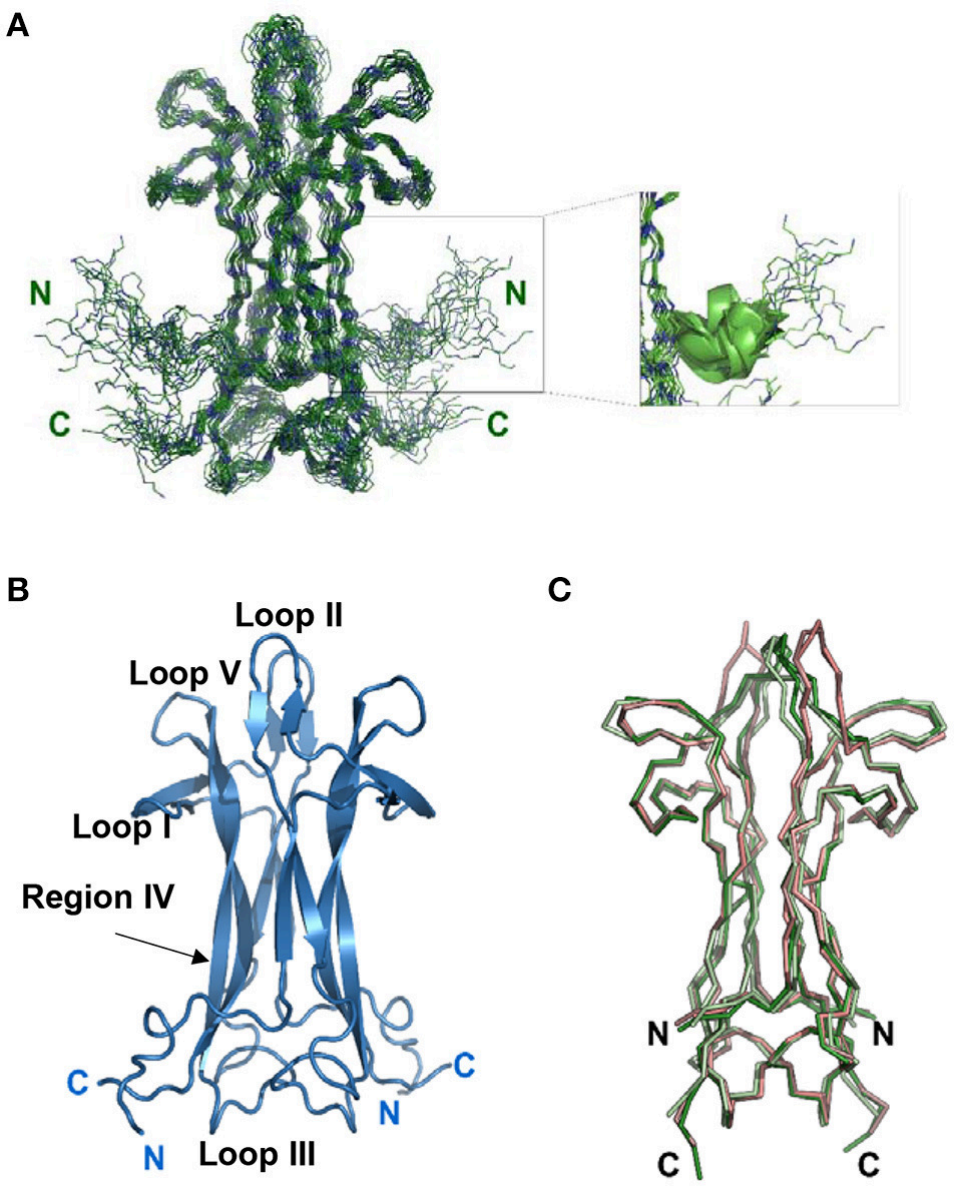
**Solution structure of recombinant mNGF**. **(A)** Overlay of the 20 lowest global energy models after ARIA refinement in water. Inset: Subgroup of 11 structures with a nascent helical structure of the N-terminus. **(B)** Cartoon model of the lowest global energy model for mNGF with the position of the loops indicated. The accepted nomenclature for neurotrophins was used (McDonald et al., 1991). **(C)** Structural alignment of the X-ray crystal structures of mNGF. They are color coded in green (1BTG, protomers **B,C**), gray (4EAX, protomers **A,B**), red (4XPJ).

Assigning the NMR spectrum to the specific protons of a protein is the prerequisite to map interactions and any conformational change. At 30°C, the 2D ^1^H -^15^N HSQC of mNGF is optimal and reveals a wide dispersion of signals characteristic of proteins with a predominantly β-sheet content which is consistent with the X-ray structure. The indole correlations of the three Trp residues are clearly observable in the distinctive and typically isolated region of the spectrum around 10.5 ppm (^1^H) and 135 ppm (^15^N). All Gln and Asn side chains are detected. We achieved virtually full assignment of the spectrum. Conversion of the NOE information into a structural model was not trivial because the potential symmetry (two-fold) of the homodimer makes it hard to distinguish between intra- and inter-molecular NOEs. The problem, which has been debated for years (Saudek et al., 1991; Nilges and O'Donoghue, 1998), was circumvented thanks to the software support of ARIA (Rieping et al., 2007) which allows discrimination of intra- from inter-molecular NOEs and careful iterative analysis of the violations (Table 1). The process led eventually to a well-converged ensemble (Figure 1A) with a root mean square deviation (r.m.s.d.) of 1.3 Å from the structure with the lowest global energy as calculated on 236 residues (Figure 1B). It closely resembles the available X-ray structures, especially in the Cys-knot, while exhibiting larger variability in the loop regions (Figure 1C). The r.m.s.d. values between the structure with the lowest global energy and the crystal structures of unbound mNGF (PDB ID 1BTG, protomers B,C) or NGF bound to lipids (PDB ID 4EAX or 4XPJ) for the backbone atoms of the core residues (15–22, 51–58, 78–89, 100–111) which exclude the loops are 2.32, 2.14, and 2.12 Å respectively. The structure in solution allows us to address a number of crucial aspects as detailed in the following sections.

**Table 1:** **NMR refinement statistics of NGF**.

**COMPLETENESS OF RESONANCE ASSIGNMENTS**	
**Backbone (%)**	**97.3**
**Side Chains (%)**	**72.9**
**NMR DISTANCE AND DIHEDRAL RESTRAINTS**	
**Distance restraints**	
** Total NOEs (used during calculations—per protomer)**	**2327**
** Unambiguous**	**2315**
** Ambiguous**	**12**
** Merged**	**4331**
** Intra-residue^a^**	**1346**
** Inter-residue^a^**	**969**
** Sequential (|i–j| = 1)**	**421**
** Non-sequential (|i–j| > 1)**	**548**
** Hydrogen bonds (per protomer)**	**46**
** No. of Noe restraints per residue**	**19.9**
**Total dihedral angle restraints^b^ (per protomer)**	
** Phi angles**	**55**
** Psi angles**	**56**
**Inter-protomer restraints (per protomer)**	**33**
**STRUCTURE STATISTICS**	
**Violations**	
**Distance constraints (>0.3 Å)**	**5**
**Deviations from idealized geometry (over 60 structures)**	
** Bond lengths (Å)**	**0.0035 ± 0.00009**
** Bond Angles (°)**	**0.47 ± 0.016**
** Improper Angles (°)**	**1.46 ± 0.13**
**Ensemble RMSD (over 60 structures in the refinement process)**	
**All residues**	
** Backbone (N,CA,C,O) (Å)**	**2.58 ± 1.18**
** Heavy atoms (Å)**	**2.89 ± 1.11**
**Ordered residues^c^**	
** Backbone (N,CA,C,O) (Å)**	**1.44 ± 0.64**
** Heavy atoms (Å)**	**1.77 ± 0.57**

a *Statistics among unambiguous restraints*.

b *TALOS-derived dihedral restraints*.

c *Ordered residues (for A and B protomers): 5–6, 14, 17–24, 27–38, 41–43, 47–48, 53–58, 63, 67, 70–71, 75–82, 84–92, 97–112, 114*.

### The structure of the NGF N-terminus

From our data, we can obtain direct information on the structure of the N-terminus (residues S1-M9) from the NOE patterns. In solution, the N-terminus, whose resonances can be observed distinctly, does not adopt a regular secondary structure. However, a number of medium-range NOEs were observed between the backbone and side-chains of these residues, suggesting the existence of a short nascent helical turn (residues 6–10). The average O_i_…NH_i+3_ and O_i_…NH_i+4_ distances between residues S2-G10 were measured for the 20 lowest global energy models to check the presence of distances compatible with hydrogen bonds. Overall, the V6-G10 segment has a clear propensity to form a short helix, the O_i_…NH_i+3_ and O_i_…NH_i+4_ distances being within 2.6 and 6.1 Å over the whole bundle. In almost 50% of the 20 lowest global energy models, the V6-G10 segment adopts either a 3_10_ helix or a α-helix conformation (Figure 1A, inset). These distances are within 2.1 and 6.2 Å in the lower global energy model exhibiting a helical conformation. These results provide a clear answer to whether and how much the N-terminus is folded and clarify its tendency to fold into a helical conformation. The secondary chemical shifts, that is the difference between the observed values and the random coil values for the same amino acid, confirm a helical tendency of this region (data not shown).

### The NGF loops are in a slow conformational exchange

**Figure 2:**
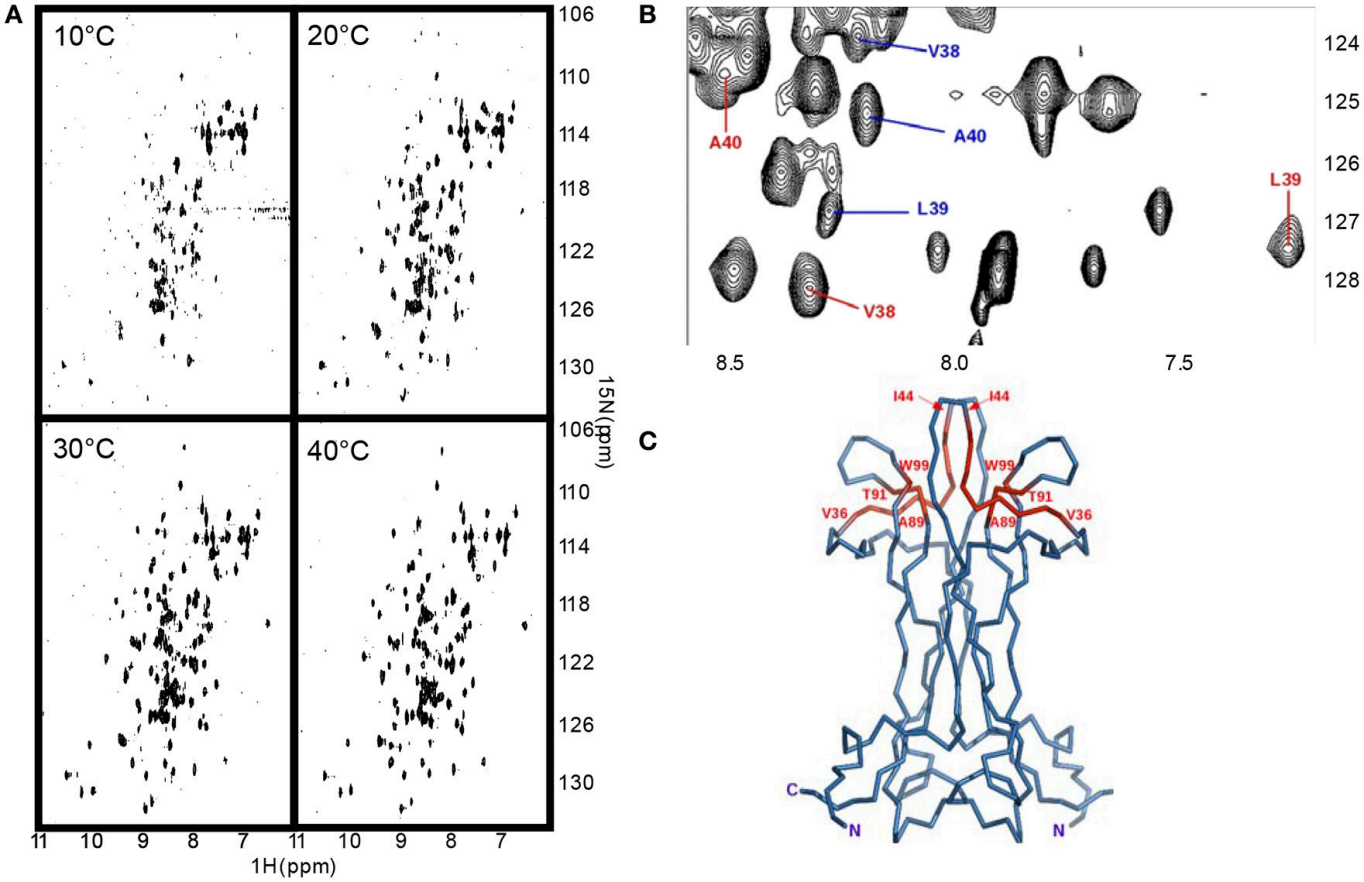
**NGF is in a conformational exchange regime. (A)** Effect of a temperature scan from 10 to 30°C on the 15N HSQC spectra of NGF. **(B)** Close-up of one of the regions of the 2D 1H-15N-HSQC at 30°C showing the presence of double species. In blue the main species, in red the secondary ones. **(C)** Mapping on NGF structure (blue) of the residues showing a double species in the spectra (red). The residues where peak splitting was observed were: V36-I44, A89-T91, W99 (amidic indole only).

While assigning the NGF spectrum which was troublesome due to significant overlap, it became clear that some interesting dynamical processes were going on. At 30°C the vast majority of the peaks have homogeneous intensity but a limited set of resonances of weaker intensity are observed (Figure 2A). After virtually full spectral assignment, these weaker resonances were found to be duplicate assignments for a small set of specific resonances which cluster around loops I and II and on the stem region (region IV-loop V) that points toward loop II (residues 39–47) (Figures 2B,C). Notably, W99, present in this region, shows doubling of the indole proton. Loop II, the region where most of the resonance splitting is observed, is also involved in small ligand binding (PDB ID 4EAX and 4XPJ). Peak doubling was furthermore observed for the side chains of a few residues in the ^13^C HCCH-TOCSY and ^13^C NOESY-HSQC spectra (data not shown). To test for the presence of conformational exchange, ^15^N HSQC spectra were recorded at 10°C intervals between 10°C and 40°C (Figure 2A). At 10°C, the peak intensity is highly heterogeneous with only ca. 30% of the number expected for a homodimer of 118 amino acids. By progressively increasing the temperature to 40°C, all the expected signals return, with the vast majority showing homogeneous intensity. However, a subset of secondary species of lower intensity remained observable. The overall quality of the spectrum is consistent with that expected for a homodimer of 26 kDa, with a line width indicative of the molecular size of the system. For a homodimeric protein, resonance doubling can be explained in at least two possible ways. It could indicate an intrinsic local asymmetry of the homodimer over the NMR timescale. This possibility can, however, be ruled out since the most affected region around loop II is not engaged in the homodimer interface. Alternatively, it could arise from the presence of multiple conformational species in equilibrium on the slow-exchange timescale. This second possibility seems more likely given the dramatic change in the number and in the relative peak intensities observed in the ^15^N HSQC as a function of temperature.

The data thus suggest that the NGF loops adopt different conformations in the slow exchange regime in the NMR timeframe.

### Dynamical features of the NGF loops

**Figure 3:**
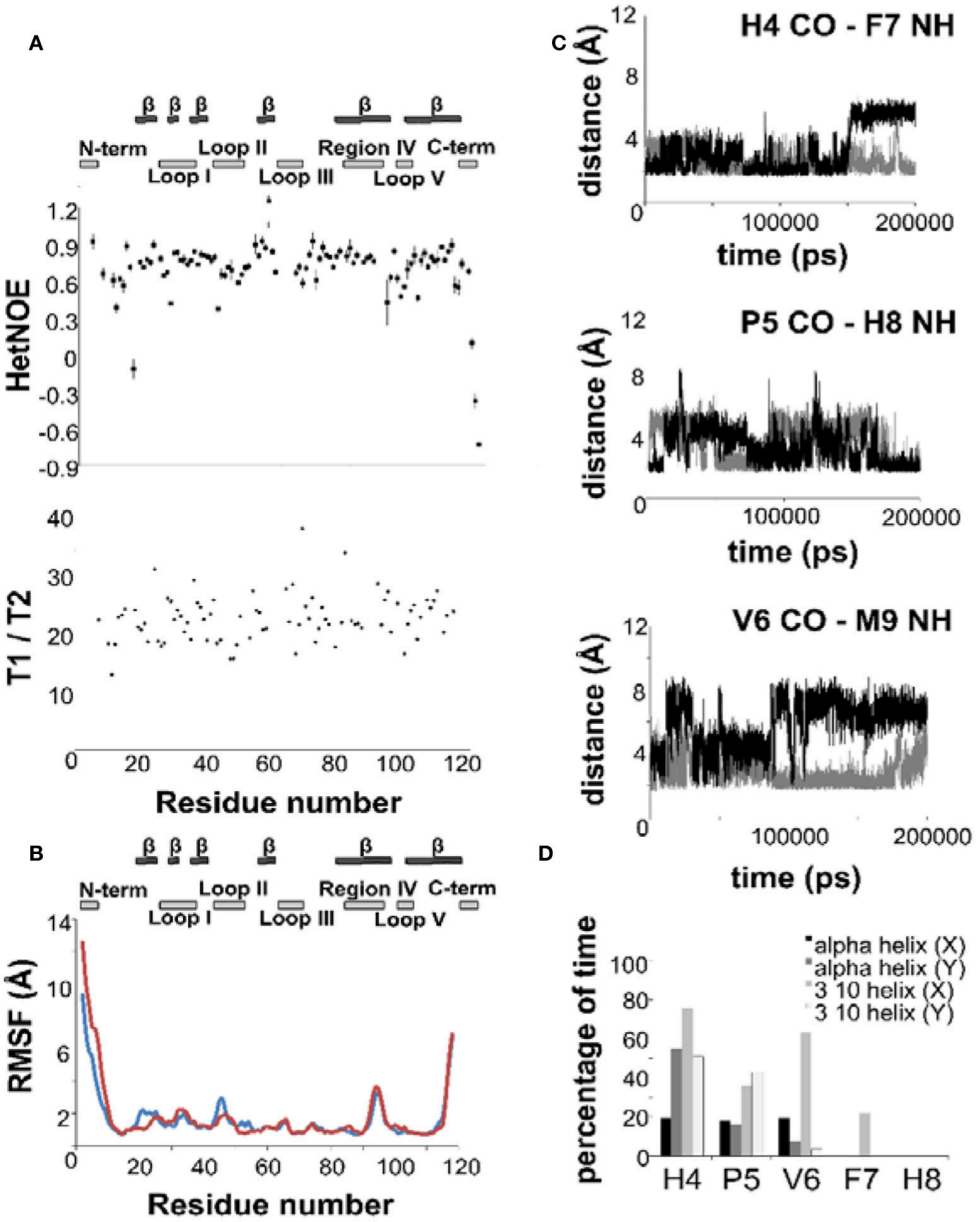
**The dynamics of NGF in solution**. **(A)** ^15^N—NMR relaxation studies. Plots of the T1/T2 and 15N-1H NOE of mNGF at 600 MHz and 30°C. The secondary structure is shown above. **(B)** R.m.s.d. fluctuations along the 200 ns MD simulation of NGF vs. residue position for the two protomers (blue—protomer x; red—protomer y). **(C)** Evolution of the distances (Å), along the 200 ns MD simulation, between atoms engaged in the hydrogen bonds that stabilize the helical structure of the N-terminus of mNGF (gray—protomer x; black—protomer y). **(D)** Percentage of time, along the 200 ns MD simulation, that each residue spent in a helical conformation along the trajectory.

We measured the T1, T2 and heteronuclear NOE (hetNOE) parameters to obtain information on the dynamics in solution. The experimental correlation time (T_C_) is 14.8 ns which is consistent with the expected molecular weight of the dimer. The T1, T2, and NOE values correlate well with the secondary structure (Figure 3A) and highlight the more rigid β-sheet regions. The loops, especially II and V, and the C-terminus are relatively more flexible showing hetNOE values lower than the average (0.7). However, overall, the hetNOE profile is relatively flat with some minor exceptions which could well be explained by residue overlap.

Intrigued by these results, we ran a molecular dynamics simulation of mNGF (200 ns) to further explore the tendency of the N-terminus to adopt a helical conformation and the conformational space of the loop regions. The ensemble of structures sampled in the MD trajectory is variable, but in line with the NMR analysis (Figure 3B). Not having imposed any symmetry constraints we see of course a different behavior in the two protomers. Over the simulation time scale, all residues in the tract His4-Phe7 spend a significant percentage of time in a 3_10_ or α-helix conformation at least in one of the protomers (Figure 3C). Residues H4-G10, in both protomers, unwind and fold during the simulation but are able to form transient hydrogen bonds mostly between the carbonyl oxygen of H4 and the amide hydrogen of F7 and the carbonyl oxygen of P5 and the amide hydrogen of H8. The carbonyl oxygen of V6 is also engaged only in one protomer in hydrogen bonds with the amide hydrogen of either M9 or G10 residues. The main-chain–side-chain interactions between residues H4/F7, V6/M9, and F7/G10 are overall more preserved than the side-chain–side-chain interactions between residues P5/H8 and V6/M9 as well as the interaction between the residues V6/G10. The MD results are in line with the observed NOE contacts supporting that in at least one of the protomers (Table S1), a short segment within the central region of the mNGF N-terminus adopts a α-helix (15%) or a 3_10_-helix (35%) conformation for a small but significant fraction of time (Figure 3D). According to these conclusions, a previous MD study on an isolated linear peptide spanning the N-terminal sequence (Stanzione et al., 2010) has shown that most of the adopted conformations do not present a regular secondary structure but a helical structure can form for a limited fraction of conformers in the ensemble.

We can thus conclude that the NGF N-terminus comprises a nascent helix which can be further stabilized upon binding with its partners.

### Analysis of the loop motions

The C_α_ r.m.s.d. along the MD trajectory shows that, besides the N- and C-termini, the regions encompassing loops II and V are comparably more flexible and exhibit large-scale conformational flexibility (Figure 3B). The loop variations are however relatively small as compared to the flexibility of the N- and C-termini. This indicates that the loops are plastic but not flexible in agreement with the differences observed in these regions in the crystallographic structures of apo mNGF (PDB ID 1BET, 1BTG) and in complex with lysophosphatidylserine (PDB ID 4EAX) and lysophosphatidylinositol (PDB ID 4XPJ). This information is of crucial importance for the development of small organic compounds (Brahimi et al., 2014) and peptidomimetics based on the combination of different NGF loop fragments (Colangelo et al., 2008). We thus analyzed the relative intra- and inter-protomer distances between loops I, II, III, and V (Figure S1). The shortest and the longest inter-protomer C_α_ distances between the Asn46 of Loop II are 11 Å (apo form PDB ID 1BTG, protomers B,C) and 30 Å respectively (holo forms PDB ID 4XPJ, protomers A,B and PDB ID 4EAX, protomers A,B and C,D) indicating large scale motions. The corresponding distances, in the most representative MD structure (the most populated cluster center) and in the lowest global energy NMR structure are 13 and 16 Å respectively. The longest and shortest inter-protomers distances between Lys95 of Loop V are 42 Å (apo form PDB ID 1BTG, protomers B,C) and 40 Å (holo form PDB ID 4XPJ). The corresponding distances in the most representative MD structure (the most populated cluster center) and in the lowest global energy NMR structure are 34 and 37 Å respectively (Figure S1).

For comparison, in the crystal structures of free and bound NGF, the conformation of loop II has high plasticity with an overall opening of the structure upon the accommodation of the small ligands (Figure 1C). It is tempting to speculate that the conformational equilibrium experienced by loop II and V, supported also by resonance doubling, is functionally relevant for the biological activity of the molecule, including interactions with the binding partners, i.e. the receptors TrkA and p75^NTR^. These features highlight the differences between NGF and other homodimeric β-sandwich structures.

We can thus conclude that NGF has long plastic but relatively rigid loops. This information is of crucial importance for drug design (Bannwarth and Kostine, 2014).

### Using the spectral assignment for antibody recognition studies

**Figure 4:**
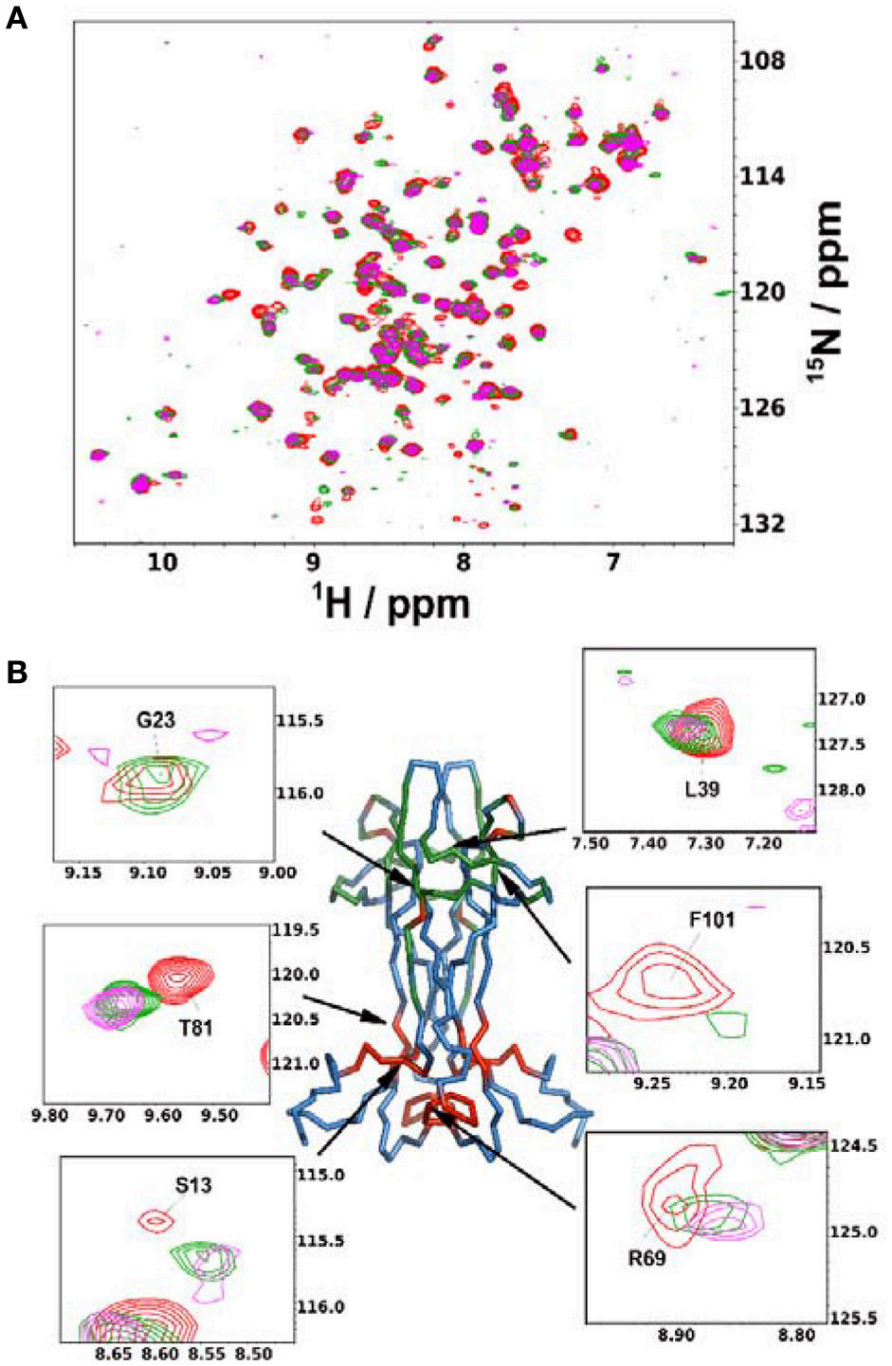
**Anti-NGF Antibody-antigen interaction. (A)** Superimposition of the ^15^N-HSQC recorded at the different points of the titration of mNGF with the MAb αD11. The spectra were recorded at 30°C. Red: 0 antibody equivalents; Green: 0.15 antibody equivalents; Purple: 0.45 antibody equivalents. **(B)** Structure of mNGF (PDB ID 1BET) with the residues of the αD11 epitope highlighted in green. Residues found to be affected in the present titration but not previously known to participate to the epitope are highlighted in red. Insets: Close-ups of the residues most affected by broadening or chemical shift perturbation. Their positions on the structure is indicated by arrows.

Finally, we used the information gained in solution to study the interaction of NGF with αD11, one of the well-characterized antibodies. We titrated ^15^N-labeled NGF with αD11 using the MAb protein. We used sub-stoichiometric amounts of αD11 to allow the observation of shift changes, given that the high affinity expected and the large molecular weight of the complex could prevent observation of the signals at stoichiometric concentrations (Figure 4A). The most appreciable perturbations in the NGF spectrum occur in three distinct sets of resonances which disappear (V14, G23, F101, C110, V111), decrease in intensity (T26, L39, N45, N46, Y52, E65, D93, W99, R100, K115) or shift significantly (G10, S13, V18, A40, F49, T56, Y79, T81, T92, E94, K95, Q96, I102, V109) indicating slow, slow-intermediate and fast-exchange regimes, respectively. When mapping these regions onto the NGF structure, a set of the affected residues (V18, G23, T26, L39, A40, F49, Y52, D93, Q96, W99, R100, F101) overlap with those previously identified in the SAXS hNGF-αD11 FAb complex model (Covaceuszach et al., 2008). Additionally, residues G10-V14, V64-I71, T81, and V109-V111, which cluster close together in space, suggest a previously unidentified epitope (Figure 4B). Interestingly, this region is not included in the FAb-NGF contacts in the crystal structures of the complexes of hNGF with the anti-NGF antibodies Tanezumab (PDB ID 4EDW) and its murine precursor MAb 911 (PDB ID 4EDX), whose epitopes are otherwise comparable to those of αD11 FAb. Thus, the solution NMR epitope mapping of the MAb αD11 unveiled a previously undetected epitope, further supporting why αD11 antibody binding differs significantly between mature NGF and proNGF: in the latter the pro-peptide could hinder the accessibility of the NGF surface to the antibody.

## Discussion

NGF is an important molecule involved in the maintenance of numerous neuronal populations, and its alterations in metabolism are crucial for different neurological disorders. The crystal structures of mNGF have been known for 25 years (McDonald et al., 1991; Holland et al., 1994), but no solution structure has so far been available which could facilitate drug design and small molecule screening in solution. The present study fills this gap, by reporting the mNGF structure in solution and sheds light on local conformational and dynamical features supported by MD simulations.

For the first time we have experimentally characterized the N-terminus of the protein, a dynamic and elusive segment of NGF which was previously only observed in the structure of recombinant hNGF in complex with TrkA receptor while it is cleaved or unobservable in other structures. This region is considered a structural determinant in NGF molecular recognition and selective binding of the TrkA rather than p75^NTR^ receptor (Wiesmann et al., 1999; He and Garcia, 2004; Wehrman et al., 2007). Our data indicate that, in the context of the full length mNGF, a helical conformation is readily sampled in solution among the energetically favored states of this region as in a classical nascent helix. This result challenges the current view that the NGF N-terminus is unstructured and adopts a helical structure only upon binding to its partner TrkA (Wehrman et al., 2007). Our findings open up new strategies for the development of effective NGF N-terminal based bioactive peptide-based compounds as antagonists with high-affinity toward the TrkA receptor.

From a careful analysis of the resonances in the ^15^N- and ^13^C-HSQC and the corresponding NOESY-HSQCs, it was also possible to identify the splitting of several resonances. The residues involved are all concentrated in the region of the molecule encompassing loops I and II and the facing region IV/loop V. The occurrence of these extra resonances likely results from a slow exchange between conformational species, thus pointing to an increased mobility of the loops II and V within the frame of an otherwise rigid molecule. Accordingly, these regions have relatively lower values of hetNOEs and a higher r.m.s.d. in our MD trajectory although overall the motions look only local. These observations agree with the structural information available for other members of the neurotrophin family (Butte et al., 1998; Robinson et al., 1999; Banfield et al., 2001; Gong et al., 2008) and with the observation that the region encompassing loops II and V is the one in which a conformational rearrangement/breathing is triggered by binding of lysophospholipids (Tong et al., 2012; Sun and Jiang, 2015). We can rationalize these results considering that loops II and V can be considered like forceps that open/close and are in a cross-talk to adapt and sequester the ligand. Loops I are spatially close to loops V but do not move. They could thus be considered like a “guard rail” preventing excessive motions of loops V and consequent disruption of the binding site formed by loops II and V. Loop III protects the cystine-knot. It is spatially far from loops I, II and V, but in proximity of the N- and C-termini. It is not flexible in agreement with its likely role of protection even though it is peculiar that this loop has partially defined electronic density in the crystallographic structures of human NGF in complex with the TrkA and p75^NTR^ receptors, as well as in complex with the DNA-aptamer and with antibodies and it is among the least conserved in length and composition within the neurotrophin family members (NGF, BDNF, NT-3, NT-4).

Finally, the present NMR study exploits the interaction between NGF and the MAb αD11 antibody allowing a fine mapping of the epitope: We observed a patch encompassing residues G10-V14, V64-I71, T81, and V109-V111, as being involved in addition to loop I and II which had already been identified (Covaceuszach et al., 2008). These regions face each other in the structure. The observation is important for the interpretation of the binding properties of αD11 which recognizes both mature and proNGF albeit with very different affinities (pM *vs*. nM). Since αD11 is an NGF neutralizing antagonistic antibody (Covaceuszach et al., 2008), knowledge of the exact epitopes is of interest for interpreting mouse models and for the design of more effective antibodies.

In conclusion, our study reports the first NMR study of mNGF in solution, and, more generally, of a neurotrophin and provides new insights into the dynamical features of NGF N-terminus and loops, opening the way to further studies aimed at a deeper understanding of NGF binding to its partners. It also paves the way to the determination of the high resolution structure of the mNGF precursor, mproNGF, whose only available 3D structural information is limited to low resolution SAXS studies (Paoletti et al., 2009) and preliminary solution NMR studies (Paoletti et al., 2011).

### Experimental procedures

#### Expression, refolding, and purification of mNGF

Recombinant mNGF was expressed in minimal medium, both as ^15^N and a ^15^N-^13^C labeled proteins, and purified as previously described (Paoletti et al., 2011). See Supplementary Information for details.

#### NMR spectroscopy experiments

NMR experiments and structure calculations were carried out using standard protocols (details in Supplementary Information). Briefly, HNCA, HNCO, HNCACB were used for backbone assignments. ^15^N NOESY-HSQC, ^13^C NOESY-HSQC, CBCACONH, and HCCH-TOCSY were used for side chain aliphatic assignments. (Hβ)Cβ(CγCδ)Hδ and (Hβ)Cβ(CγCδ)Hε were used in combination with ^13^C-HSQC, ^13^C- NOESY-HSQC, and HCCH-TOCSY tuned for the aromatic resonances for the assignment of the aromatic side chains. All spectra were processed using NMRPipe/NMR-Draw (Delaglio et al., 1995) and analyzed using CARA (Keller, 2004). Backbone and side chain assignment was deposited in the BMRB database (accession code 34037).

Relaxation parameters were obtained by spectra recorded at 600 MHz and 30°C and extracted using peak picking, lineshape fitting, and exponential modeling as implemented in NMRPipe (Delaglio et al., 1995).

#### Structure determination

Automated NOESY cross-peak assignments and structure determination were performed using the ARIA 2.3 software (Rieping et al., 2007). The input used to generate the final structures consisted of NGF intra-molecular NOE cross peaks from ^15^N- and ^13^C-NOESY-HSQC spectra (at 30 and 35°C), along with a set of φ and ψ backbone dihedral restraints derived by TALOS+ (Shen et al., 2009) and a set of manually assigned unambiguous inter-protomer restraints. After refinement of the 60 lowest global energy structures by molecular dynamics simulation with explicit water, 20 structures ranked on global energy were selected as representative of the structure and used for statistical analysis. Structure quality was evaluated with PROCHECK-NMR (Laskowski et al., 1996). The details are reported in the Supplementary Information.

The coordinates of the NGF structure are deposited in the PDB under the code 5LSD.

#### Molecular dynamics simulations and analysis

A molecular model of the full length mNGF encompassing residues (2–118) was built (Covaceuszach et al., 2015) from the crystal structures of mouse bis-*des-octa* β-*NGF* (PDB ID 1BTG, protomers B,C). Molecular dynamics simulation (MD) was performed using the GROMACS software package (version 5.1.2) (Hess et al., 2008) conjugated with the Amber99SB force field. Details are reported in the Supplementary Experimental procedures. The utilities *gmx distance* and *gmx cluster*, provided in the GROMACS package, were used: (i) to calculate the distances between pairs of positions as a function of time; (ii) to cluster, in the post processing phase, the resulting trajectories with a cutoff of 0.15 nm, calculated on the backbone atoms (Daura et al., 1999).

## Author contributions

FP, CdC, DL, AP, AC conceived and designed the experiments; FP, CdC, FM, RY, SC, GK conducted the experiments and analyzed the data; FP, CdC, DL, AP, AC interpreted the data and wrote the paper.

## Funding

The research described here was funded by the European Community's Seventh Framework Program Paincage Grant Nr 603191, MRC (U117584256), and MIUR, project PRIN #2010N8PBAA_006. FP was recipient of a Royal Society—Accademia dei Lincei Fellowship.

### Conflict of interest statement

The authors declare that the research was conducted in the absence of any commercial or financial relationships that could be construed as a potential conflict of interest.
