# Supplementary material for: Conformational Rigidity within Plasticity Promotes Differential Target Recognition of Nerve Growth Factor
Source: Front Mol Biosci. 2016 Dec 26;3:83. doi: 10.3389/fmolb.2016.00083 (PMC5183593; doi:10.3389/fmolb.2016.00083)

Supplementary Experimental Procedures

Expression, refolding and purification of mouse NGF

mNGF heterologous expression was performed in BL21(DE3) E. coli transformed with the plasmid pET11a containing the gene of mouse proNGF25 (Paoletti et al., 2009). Solubilization and refolding of proNGF25 from inclusion bodies was carried out according to (Rattenholl et al., 2001). Mature NGF was obtained following enzymatic proteolytic cleavage of proNGF25 by trypsin (Paoletti et al., 2009). The protein fold was characterized by biophysical techniques as previously reported (Paoletti et al., 2009, 2011).

NMR spectroscopy experiments

NMR spectra for structural determination were acquired on samples containing ^{15}N- or ^{15}N,^{13}C-labelled NGF (0.1 mM) in 50 mM sodium phosphate at pH 7 with 1 mM EDTA and 8% ^2H_2O. The spectra were recorded at 30°C using Varian Inova spectrometers operating at 600 and 800 MHz ^{1}H frequency, the 800 MHz being equipped with a triple resonance gradient cryo-probe, and Bruker Avance spectrometers operating at 600 and 700 MHz ^{1}H frequency, both equipped with triple resonance gradient cryo-probes. HNCA, HNCO, HNCACB experiments were employed to obtain sequence specific ^{1}HN, ^{15}N, ^{13}Cα, ^{13}Cβ and ^{13}C backbone assignments (Muhandiram and Kay, 1994).

Side chain aliphatic proton and carbon assignments were achieved by a combination of 3D ^{15}N-edited NOESY-HSQC, ^{13}C NOESY-HSQC, CBCACONH and HCCH-TOCSY.

(Hβ)Cβ(CγCδ)Hδ and (Hβ)Cβ(CγCδ)Hε experiments were used in combination with ^{13}C-HSQC, ^{13}C- NOESY-HSQC and HCCH-TOCSY tuned for the aromatic resonances for the assignment of the large number of the aromatic side chains.

All spectra were processed using NMRPipe/NMR-Draw (Delaglio et al., 1995) and analyzed using CARA (Keller, 2004). Intra-molecular proton distance restraints were derived from ^{15}N- and ^{13}C-NOESY-HSQC spectra (mixing time 100 ms). A second set of ^{15}N- and ^{13}C-NOESY-HSQC spectra was recorded at 35°C. Backbone assignment was guided by the identification of the expected set of connectivities within and inter-strands, while side chains assignment was complicated by the severe overlap of resonances from Leu, Ile, Lys, Arg residues which are highly abundant. Several of these resonances correspond to residues which cluster at the homodimeric interface. Assignment of the aromatic side chains could be obtained almost to completeness, although limited

ambiguity remains for the side chains of some phenylalanines (Phe7, Phe12, Phe49). Backbone and side chain assignment was completed at 98% and 80%, respectively.

Relaxation parameters were obtained by spectra recorded at 600 MHz and 30°C, with ^{1}H spectral width 16.7 ppm, ^{15}N spectral width 30 ppm, acquisition times of 102 ms (^{1}H) and 46.6 ms (^{15}N). T_1 spectra were recorded with delays of 10, 100, 300, 500, 800, 1100, 1600 ms. T_2 spectra were recorded with delays of 8, 16, 24, 32, 48, 64, 80, 96 ms. HetNOEs were recorded with a saturation time of 5 seconds. Relaxation parameters were extracted using peak picking, lineshape fitting, and exponential modelling as implemented in NMRPipe (Boyd et al., 1990; Kay et al., 1989).

Structure Determination

Automated NOESY cross-peak assignments and structure determination were performed using the ARIA 2.3 software (Rieping et al., 2007) based on an almost complete assignment of NGF and on a large number of intra- and inter-molecular NOEs. The ARIA input used to generate the final structures consisted of NGF intra-molecular NOE cross peaks from ^{15}N- and ^{13}C-NOESY-HSQC spectra (at 30°C and 35°C), along with a set of φ and ψ backbone dihedral restraints derived by TALOS+ (Shen et al., 2009). The NMR structural bundle was generated by ARIA using ambiguous and unambiguous intra-protomer distance restraints (both manually and automatically assigned) derived from ^{15}N- and ^{13}C- NOESY-HSQC (aliphatic and aromatic) experiments at 30°C and 35°C, in addition to a set of manually assigned unambiguous inter-protomer restraints. The total number of unambiguous intramolecular restraints used in the calculations was 2327 (per protomer) plus 33 intermolecular (per protomer). Dihedral restraints derived by TALOS+ (55 phi/psi couples/protomer) and secondary structure-derived H-bonds (46/protomer) were also used as restraints, the latter only being introduced when present in 50% the preliminary calculations. In the last ARIA run, 200 structures were generated in the final iteration. After refinement of the 60 lowest global energy structures by molecular dynamics simulation in water, 20 structures were selected as representative of the structure and used for statistical analysis. Structure quality was evaluated with PROCHECK-NMR (Laskowski et al., 1996).

Molecular Dynamics Simulations and Analysis

A molecular model of the full length mNGF encompassing residues (2-118) was built

(Covaceuszach et al., 2015) from the crystal structures of murine bis-*des-octa* β-*NGF* (PDB ID 1BTG, protomers B,C). The missing N-term region (Ser 2 – Met 9) was extended by molecular modeling on the basis of the crystallographic structure of hNGF from hNGF-TrkA complex (PDB ID 2IFG, protomers E,F). Molecular dynamics simulation (MD) was performed using the GROMACS software package (version 5.1.2) (Hess et al., 2008) conjugated with the Amber99SB force field. The protein was immersed in a cubic box with periodic boundary conditions and was solvated with explicit Single Point Charge waters (Berendsen et al., 1981). The protonation state of the ionisable groups of the protein was set according to pH 7.0 and the overall charge of the system was neutralized by adding an appropriate number of counter ions. The box dimensions (9.7 nm × 9.7 nm × 9.7 nm) were set to allow at least 1.2nm between the protein and the box faces on each side. The final system consisted of 3642 protein atoms surrounded by 11,000 water molecules. Before starting the production run, the system (protein and water) was energy minimized by using the steepest descent method until convergence (1000 iterations). Next, temperature and pressure were pre-equilibrated by short 50 ps canonical NVT ensemble and 1 ns isothermal-isobaric NPT ensemble runs with restraints on the solute. A 200 ns production MD was performed by coupling the system to an external temperature bath at 300 K and an external isotropic pressure bath at 1 bar, using a time step of 0.002 ps and the trajectories were saved at each 2 ps.

All the bond lengths were constrained to their equilibrium values using the LINCS algorithm (Hess et al., 1997) for the protein. Long-range electrostatic forces were treated using the Fast Particle-Mesh Ewald method (PME) (Essmann et al., 1995). Van der Waals forces were treated using a cut-off of 1.0 nm.

Supplementary References

Berendsen, H.J.C., Postma, J.P.M., van Gunsteren, W.F., and Hermans, J. (1981). Interaction models for water in relation to protein hydration. In Intermolecular Forces, (B. Pullman ed., Reidel, Dordrecht), pp. 331–342.

Boyd, J., Hommel, U., and Campbell, I.D. (1990). Influence of cross-correlation between dipolar and anisotropic chemical shift relaxation mechanisms upon longitudinal relaxation rates of 15N in macromolecules. Chem. Phys. Lett. *175*, 477–482.

Covaceuszach, S., Konarev, P.V., Cassetta, A., Paoletti, F., Svergun, D.I., Lamba, D., and Cattaneo, A. (2015). The conundrum of the high-affinity NGF binding site formation unveiled? Biophys. J. *108*, 687–697.

Delaglio, F., Grzesiek, S., Vuister, G.W., Zhu, G., Pfeifer, J., and Bax, A. (1995). NMRPipe: a multidimensional spectral processing system based on UNIX pipes. J. Biomol.

NMR *6*, 277–293.

Essmann, U., Perera, L., Berkowitz, M.L., Darden, T., Lee, H., and Pedersen, L.G. (1995). A smooth particle mesh Ewald method. J. Chem. Phys. *103*, 8577–8593.

Hess, B., Bekker, H., Berendsen, H.J.C., and Fraaije, J.G.E.M. (1997). LINCS: A linear constraint solver for molecular simulations. J. Comput. Chem. *18*, 1463–1472.

Hess, B., Kutzner, C., van der Spoel, D., and Lindahl, E. (2008). GROMACS 4: Algorithms for Highly Efficient, Load-Balanced, and Scalable Molecular Simulation. J. Chem. Theory Comput. *4*, 435–447.

Kay, L.E., Torchia, D.A., and Bax, A. (1989). Backbone dynamics of proteins as studied by 15N inverse detected heteronuclear NMR spectroscopy: application to staphylococcal nuclease. Biochemistry (Mosc.) *28*, 8972–8979.

Keller, R. (2004). The Computer Aided Resonance Assignment Tutorial.

Laskowski, R.A., Rullmannn, J.A., MacArthur, M.W., Kaptein, R., and Thornton, J.M. (1996). AQUA and PROCHECK-NMR: programs for checking the quality of protein structures solved by NMR. J. Biomol. NMR *8*, 477–486.

Muhandiram, D.R., and Kay, L.E. (1994). Gradient-Enhanced Triple-Resonance Three-Dimensional NMR Experiments with Improved Sensitivity. J. Magn. Reson. B *103*, 203–216.

Paoletti, F., Covaceuszach, S., Konarev, P.V., Gonfloni, S., Malerba, F., Schwarz, E., Svergun, D.I., Cattaneo, A., and Lamba, D. (2009). Intrinsic structural disorder of mouse proNGF. Proteins *75*, 990–1009.

Paoletti, F., Malerba, F., Kelly, G., Noinville, S., Lamba, D., Cattaneo, A., and Pastore, A. (2011). Conformational plasticity of proNGF. PloS One *6*, e22615.

Rattenholl, A., Lilie, H., Grossmann, A., Stern, A., Schwarz, E., and Rudolph, R. (2001). The pro-sequence facilitates folding of human nerve growth factor from Escherichia coli inclusion bodies. Eur. J. Biochem. FEBS *268*, 3296–3303.

Rieping, W., Habeck, M., Bardiaux, B., Bernard, A., Malliavin, T.E., and Nilges, M. (2007). ARIA2: automated NOE assignment and data integration in NMR structure calculation. Bioinforma. Oxf. Engl. *23*, 381–382.

Shen, Y., Delaglio, F., Cornilescu, G., and Bax, A. (2009). TALOS+: a hybrid method for predicting protein backbone torsion angles from NMR chemical shifts. J. Biomol. NMR *44*, 213–223.

Table S1 – Summary of NOE of the main-chain/side-chain contacts among residues of the N-terminus and corresponding distances in the MD

Atom of first residue	Atom of second residue	Min - Max distance over MD timescale (Å)	Comment
HB His4	HA Val6	4.54-8.92	During the whole MD timescale, in contact for a variable amount of time
HB His4	HG1 Val6	2.69-10.43	For half time of the whole MD timescale close contact, then they move far apart
HD2 His4	HB Val6	2.37-11.50	During the whole MD timescale, contact for a variable amount of time
HD2 His4	HG1 Val6	2.08-12.16	For half time of the whole MD timescale close contact, then they move far apart. The two protomers behave differently
HB His4	HN Phe7	1.87-8.95	For the whole MD timescale, close contact
HA Pro5	HB Phe7	4.08-9.25	Close in space in only one of the two protomers, and for a limited amount of time
HB Pro5	HB His8	2.36-13.27	Close in space in only one of the two protomers for the part of the timescale
HA Val6	HA Met9	3.19-11.94	Close in space in only one of the two protomers, and for a limited amount of time
HG1 Val6	HN Met9	4.64-13.25	Close in space in only one of the two protomers, and for a limited amount of time
HG1 Val6	HG Met9	2.30-16.76	Close in space in only one of the two protomers, and for a limited amount of time
HB His8	HA Gly10	3.64-10.12	Close in space in only one of the two protomers, and for a limited amount of time
HA Gly10	HE Phe12	2.07-11.12	Great distance variability during time, in both protomers.

Figure S1 – Superimposition of NGF structures.

Panel A – Superimposition of X-ray crystal structure of mNGF (PDB ID 1BTG, protomers B,C – green), NMR lowest global energy structure (blue) and MD more representative cluster (purple). Two different views are shown.

Panel B – Close-up of the loops II and V in the three structures

The figures were generated using PyMol (www.pymol.org)

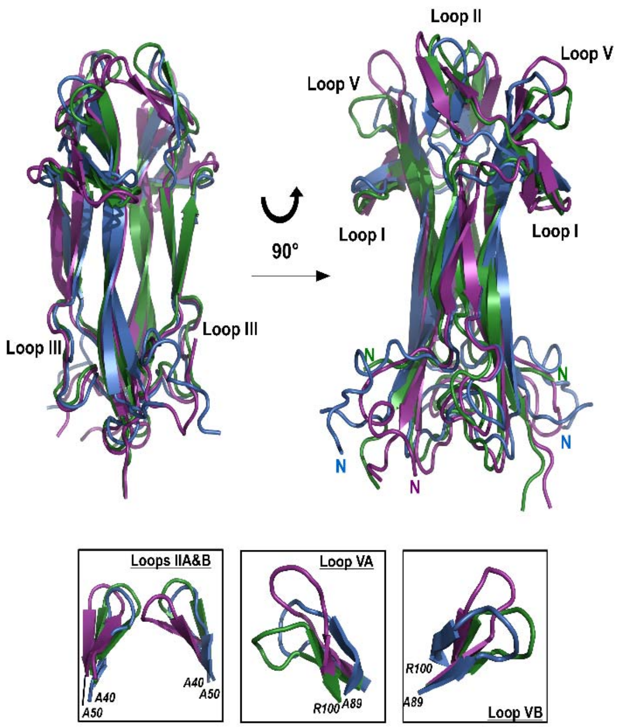

Supplement: Supplementary file 1 [file DataSheet1.pdf]
